# Interplay between CO and Surface Lattice Oxygen Ions in the Vacancy-Mediated Response Mechanism of SnO_2_-Based Gas Sensors

**DOI:** 10.1021/acssensors.4c03047

**Published:** 2025-03-01

**Authors:** Stefan Kucharski, Michael Vorochta, Lesia Piliai, Andrew M. Beale, Christopher Blackman

**Affiliations:** † Department of Chemistry, University College London, 20 Gower St, London WC1H 0AJ, U.K.; ‡ Research Complex at Harwell, Rutherford Appleton Laboratory, Harwell, Didcot OX11 0FA, U.K.; § Department of Surface and Plasma Science, Faculty of Mathematics and Physics, Charles University, V Holešovičkách 2, Prague 8 180 00, Czechia

**Keywords:** conductometric, mechanism, NAP-XPS, resistance, sensor, spectroscopy, vacancy

## Abstract

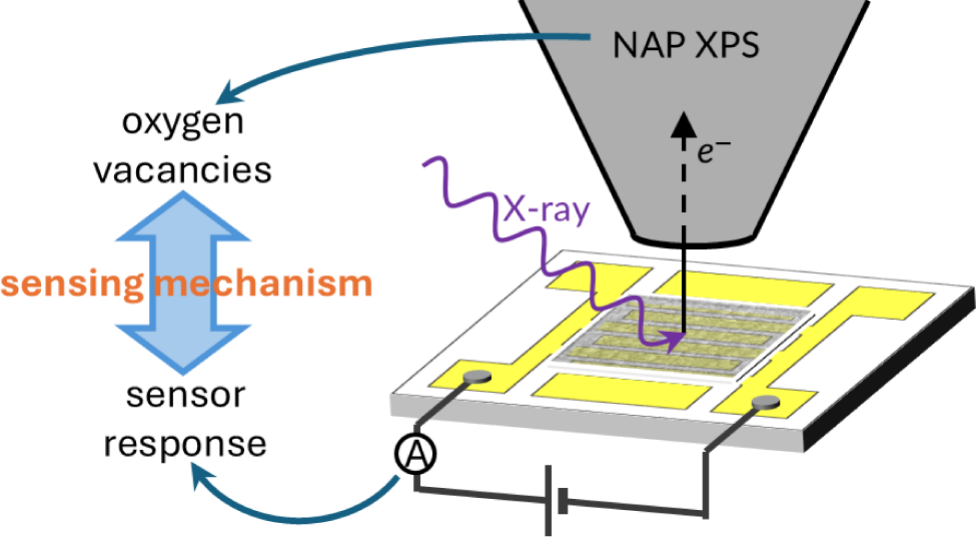

Despite having been commercially available for more than half a century, conductometric gas sensors still lack a definite description of their operation mechanism, which hinders research into improving their characteristics. With the advent of operando spectroscopy comes the opportunity to elucidate their working principle by observing their surface during sensing. To that end, we have employed near-ambient pressure (NAP) XPS with simultaneous resistance measurements to correlate the macroscopic sensor response with atomistic changes to the sensor’s surface under exposure to CO, a common target gas. Our results show a clear relationship between the sensor response and the change in surface stoichiometry of SnO_2_, suggesting that near-surface oxygen vacancies play a vital role in the sensing mechanism, in support of a vacancy-modulated “surface conductivity” mechanism.

Carbon monoxide detection has been one of the primary applications of SnO_2_-based conductometric gas sensors (CGS) since their development in the 1970s.^1^ However, despite decades of extensive research,^2−9^ the precise detection mechanism is still under debate, limiting progress toward more effective sensors. Although the improvement of sensor characteristics can be pursued by, for example, employing intricate nanostructures,^10^ metal nanoparticle decoration,^11,12^ or SnO_2_-graphene oxide (GO) composites,^13,14^ a complete understanding of the processes underlying gas detection is essential in enabling a design-led approach to new sensitive material development.

Early attempts at explaining the gas-surface interactions of SnO_2_ involved charged oxygen adsorbates, first proposed by Hauffe in 1955.^15^ The resulting “ionosorption” model is built around monatomic oxygen adsorbates O^–^ created when atmospheric oxygen molecules dissociate through the capture of conduction electrons at the surface of SnO_2_.^16−19^ Consequently, the subsurface charge carrier depletion and accumulation of localized negative charge on the surface lead to an increased sensor resistance, typically considered the baseline for resistance change comparison for measurements conducted in air. When CO is introduced, it reacts with these hypothesized oxygen adsorbates, releasing the electrons and hence decreasing the surface charge, therefore decreasing the sensitive layer’s resistance and deviation from the baseline established in pure air.^20^ Constant-voltage interrogation of the sensor under the different atmospheres produces a measurable change in electrical current magnitude, which is used as input into Ohm’s law to calculate resistance and sensor response, the ratio of resistance during target gas exposure to the baseline resistance in pure air.

Even though “ionosorption” appears to be a convincing description of CGS operation at first glance, especially considering the sheer number of recent papers that cite it,^21−24^ there are considerable problems with this description. It does not explain the “sensor drift” (a transient change in the sensor’s baseline resistance under a constant atmosphere),^25^ it fails to account for resistance change in response to CO in an oxygen-free atmosphere,^6^ but most importantly, there is no spectroscopic evidence for the existence of the monatomic O^–^ adsorbates on the surface of SnO_2_.^26−28^ Although “ionosorption” has never been disproved, there are other proposed mechanisms of CGS operation, one of which is a vacancy-based model, herein referred to as “surface conductivity”.^29−33^

“Surface conductivity” postulates that the gas-sensitive behavior of SnO_2_ depends on the density of near-surface oxygen vacancies (V_O_). These vacancies can be formed or healed when the sensor’s surface interacts with adsorbing gases, such as O_2_ or CO, as shown in Figure 1.^5^ Given a mixture of these gases in the sensor’s environment, oxygen vacancies will be created by the oxidation of CO to CO_2_ using lattice oxygen (Figure 1; top row), and the vacancies will be healed by the ambient O_2_ (Figure 1; middle row), with rates proportional to their partial pressures. The consideration of these and other possible reactions, like the coadsorption of CO and O_2_ that leads to both CO_2_ formation and vacancy healing (Figure 1; bottom row), forms a system where surface lattice oxygen is in equilibrium with ambient oxygen.

**Figure 1:**
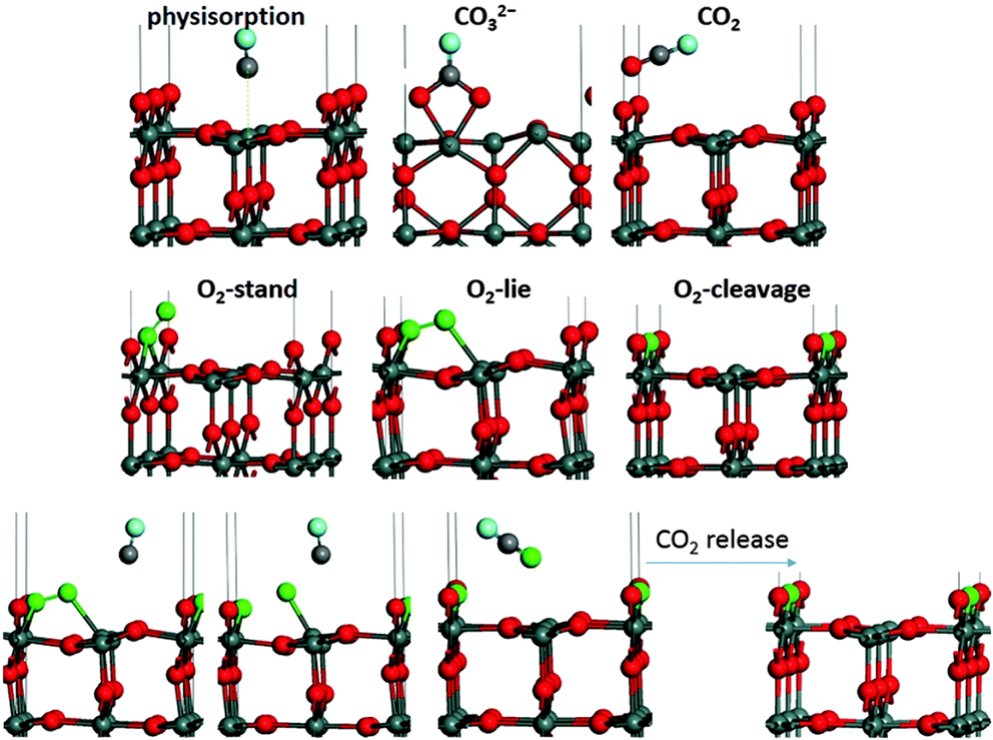
Selected examples of adsorption geometries and transition states appearing during surface reactions of SnO_2_: (top row) CO reacting with stoichiometric SnO_2_; (middle row) O_2_ reacting with substoichiometric SnO_2_; and (bottom row) CO reacting with O_2_ preadsorbed onto substoichiometric SnO_2_. Reproduced from ref. (5) with permission from the PCCP Owner Societies.

The reactions shown in Figure 1 describe the Mars-van Krevelen (MvK) mechanism of oxidation of CO on an oxide catalyst.^5^ In fact, both the catalytic oxidation and “surface conductivity” model of CO sensing are the same process observed from a different perspective—with the former focusing on the reaction products and the latter on the change in the electronic properties of the sensor (catalyst). These differences lead to an incomplete description of the full system for either catalytic or gas sensing applications, with a single unified description applicable to both gas sensing and catalysis (which often describe identical processes on identical materials) currently lacking. Obtaining a holistic picture of this process is essential in reaching an understanding that would allow a design-led approach to the development of new sensors and catalysts. In fact, the change in resistance of working oxide catalysts, including SnO_2_, in oxidation catalysis has long been noted within the catalytic community,^15^ although the “electronic theory” of catalysis has on the whole been replaced by a focus on the chemical and structural features of active sites.

Within the “surface conductivity” model, the surface V_O_, formed and healed dynamically depending on CO and O_2_ availability, are shallow electron donors that give rise to a 2D delocalized electron gas near the surface.^34^ Consequently, the surface density of charge carriers and, hence the sensor’s conductivity, are a function of the surrounding atmosphere. Therefore, any change in the gas composition that affects the equilibrium between vacancy formation and healing will, in turn, lead to a sensor response (expressed as a ratio of the resistance value when exposed to the target gas to some reference value, typically that in the absence of a target gas). Such a description of sensor operation for CO detection has recently been proposed by Degler et al., which is neatly summarized in Figure 2.^2^ This proposed mechanism is a stark departure from “ionosorption” in that it employs only spectroscopically verified species and is supported by a growing body of operando evidence obtained using, for example, infrared and UV/vis diffuse reflectance spectroscopy,^2,9^ X-ray absorption spectroscopy,^35^ and our own recent study using near-ambient pressure X-ray photoelectron spectroscopy (NAP XPS) on the interactions of SnO_2_ with O_2_.^36^ In this work, we present an analysis of the interactions of CO with SnO_2_-based CGS performed using operando NAP XPS with simultaneous resistance measurements, aiming to further clarify its mechanism of gas detection.

**Figure 2:**
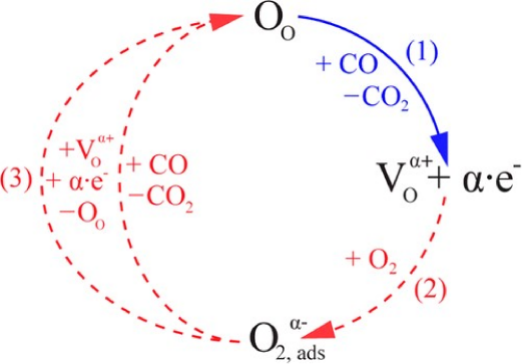
Interplay between CO, O_2_, and lattice oxygen atoms at the surface of SnO_2_ that originates the gas-sensitive behavior of this semiconducting metal oxide. Reprinted with permission from D. Degler, S. Wicker, U. Weimar and N. Barsan, *The Journal of Physical Chemistry C*, 2015, **119**, 11792–11799. Copyright 2015 American Chemical Society.

## Experimental Section

The data presented in this work were collected at Charles University, Prague, which houses a SPECS NAP XPS system equipped with a PHOIBOS 150 NAP hemispherical analyzer and a DeviSim NAP reactor cell. The XP spectra of the Sn 3d, O 1s, C 1s, and survey regions were collected at every step of the investigation. Gas flow into the reactor is regulated by mass flow controllers, and a butterfly valve maintains pressure at a prescribed level. The sample SnO_2_-based sensor, whose manufacturing has been described before,^36^ was mounted on a metal sample holder and inserted onto a heated sample stage within the NAP cell. Additionally, the sample stage was customized to allow simultaneous resistance measurements, with one electrode grounded to the sample stage and the other connected to the positive terminal of the power supply via the thermocouple lead. Because of this, the on-board thermocouple could not be used, so the temperature measurements were collected using the auxiliary thermocouple, which measures the temperature of the sample stage and correlates well with the sample’s temperature. The resistance measurements were collected using a Keithley 6517B Sourcemeter by applying a 100-mV electrical potential across the sensor’s electrodes and measuring the resulting current. The instrument was automated to collect measurements at 1 s intervals and save the results into a file; subsequently, resistance, as it is presented herein, was calculated from Ohm’s law.

This setup was used to conduct four experiments, exposing the same SnO_2_-based sensor to CO (Linde a. s., N5.0) following two different sensor pretreatments (surface reduction and oxidation) and at two temperatures (room temperature and a “high temperature” of 300 °C). In all of these experiments, the sensor was exposed to varying pressures of CO and UHV as outlined in Table 1 with XP spectra collected before CO exposure and after every pressure change.

**Table 1:** Summary of the Pressure Steps at Which XPS Data Was Collected in This Experiment (and Their Corresponding Concentration at Atmospheric Pressure)

step	before	1	2	3	after	reintro
mbar CO	UHV	0.1 mbar	0.5 mbar	1.0 mbar	UHV	0.5 mbar
ppm of CO @ 1 bar	N/A	100 ppm	500 ppm	1000 ppm	N/A	500 ppm

The first two experiments (R_RT and R_HT) were conducted consecutively on a sensor pretreated *in situ* in 5 mbar O_2_ (Linde a. s., N5.0) overnight at 500 °C, then UHV-reduced at the same temperature for 1 h and cooled down to room temperature in UHV in preparation for experiment R_RT. After collecting XP spectra at all prescribed pressure steps (Table 1), the sample was heated in UHV to 400 °C for 10 min to desorb any remaining CO and reconstitute the surface in preparation for experiment R_HT (deliberately lower than the 500 °C to avoid further surface reduction but higher than the CO desorption temperature). Subsequently, the sample’s temperature was reduced to 300 °C, and experiment R_HT commenced.

The subsequent two experiments were performed on the same sensor following an overnight *in situ* calcination in 5 mbar O_2_ at 500 °C. However, this time the sensor was not exposed to UHV following reoxidation and instead cooled to RT under the same atmosphere of 5 mbar O_2_. Once again, the room temperature experiment (O_RT) was performed first, involving the same CO pressure steps as before, followed by a surface reconstitution in 1 mbar O_2_ at 300 °C for 15 minutes, after which O_2_ was evacuated from the NAP cell and experiment O_HT commenced.

## Results and Discussion

### Interactions with a UHV-Reduced SnO_2_ Surface

#### XP Spectra Fitting

**Figure 3:**
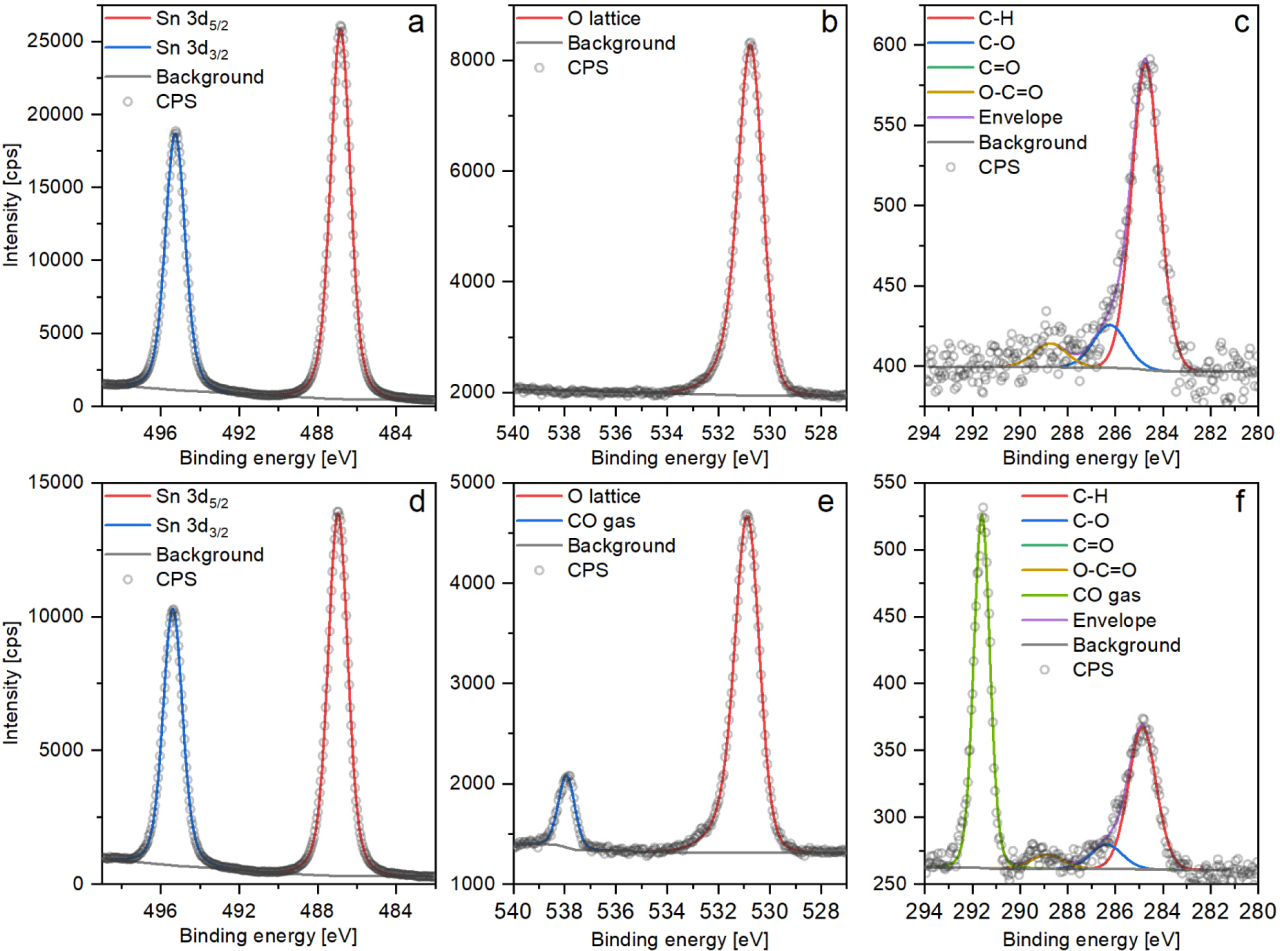
XP spectra collected on a UHV-reduced SnO_2_ surface during the room temperature CO dosing experiment: a-–c–step “before” (UHV); d–f–step ‘3′ (1 mbar of CO). The “C=O” peak in c and f is between “C–O” and “O–C=O” but hardly visible due to its small size.

**Figure 4:**
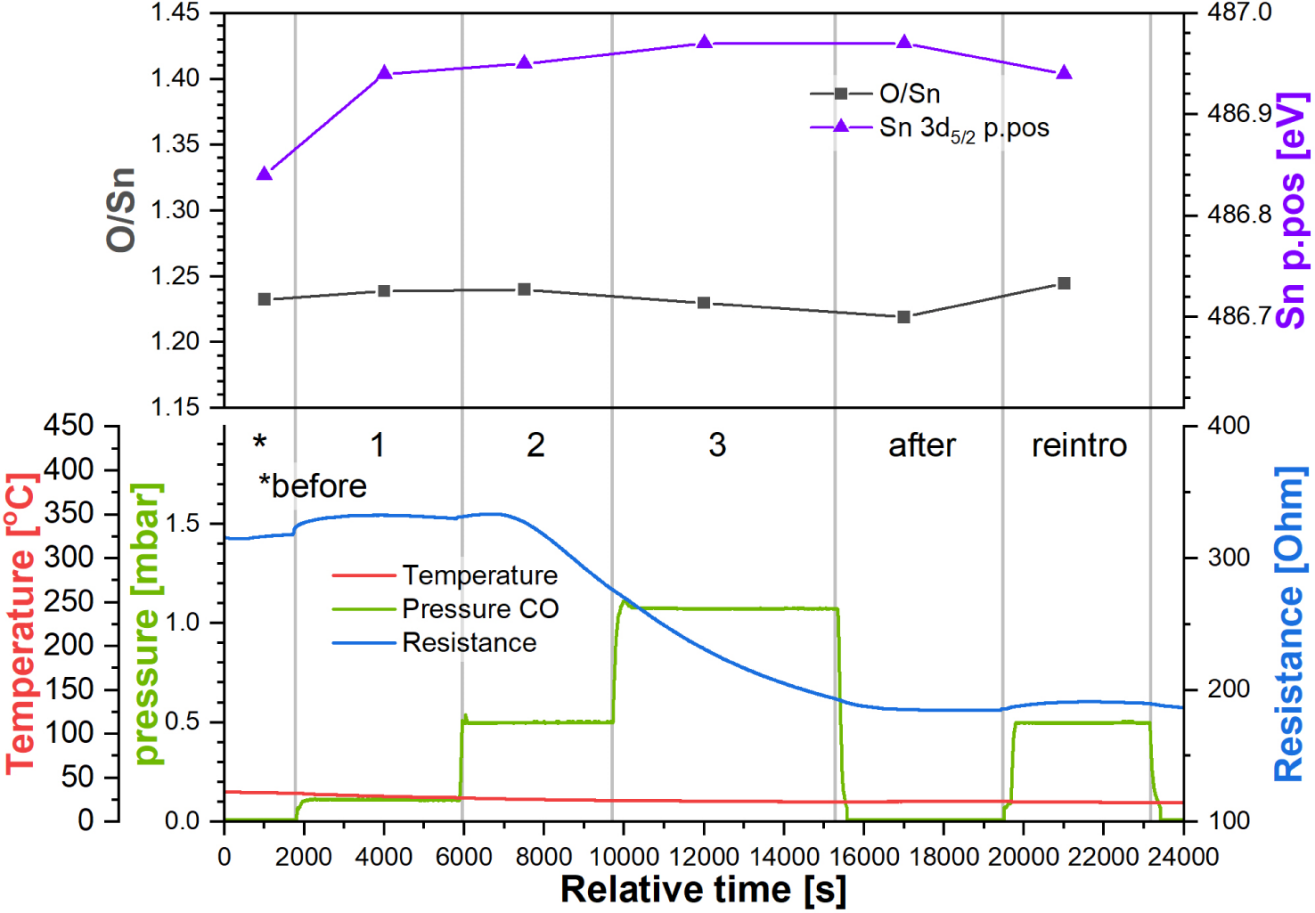
Top panel: quantification parameters derived from the Sn 3d and O 1s XP spectra collected during experiment “R_RT”. Bottom panel: temperature and pressure in the NAP cell and sensor’s resistance during experiment “R_RT”. Step labels refer to the CO pressure in the NAP cell (green trace).

Two sets of XP spectra are presented in Figure 3 as an example of the data collected on a UHV-reduced surface of SnO_2_; the remaining spectra are presented in Supporting Information. The two sets presented here were collected before CO exposure (step “before” in Figure 4) and at 1 mbar of CO (step 3 in Figure 4). The Sn 3d region is accurately reproduced using a single two-component peak that models the doublet of 5/2 and 3/2 peaks corresponding to the Sn^4+^ state^37^ (Figure 3a,d). At this point, it is essential to note that in spite of our discussing a “reduced” surface, we do not expect to see contributions from the Sn^2+^ chemical state; oxygen-deficient SnO_2_ requires significant substoichiometry before converting into an SnO phase.^38^ Instead, the electrons left after the removal of O atoms from the lattice in the surface’s plane or below it reside in the vacancies and form F-centers (in the ground state) rather than on the Sn^4+^ cation (which would transform it into Sn^2+^).^39^ While the situation is different for bridging oxygen vacancies (those protruding outward from the outermost layer of Sn atoms) where the termination of periodicity causes the electrons to localize on Sn atoms and reduce them to Sn^2+^,^5^ this change is unlikely to be observed in the Sn 3d spectrum due to the emissions from Sn^4+^ and Sn^2+^ coinciding on the binding energy scale;^40^ the reader is directed to the original text for a detailed explanation. In the O 1s region, one component models the lattice oxygen atoms^37^ (“O lattice” – Figure 3b), and the second component corresponds to the oxygen in CO molecules^41^ (Figure 3e), thus appearing only during CO exposure. The complementary C 1s scans (Figure 3c,f) were included to monitor the state of the carbonaceous contamination overlayer (originating both from exposure to ambient atmosphere and from desorption from the walls of the NAP cell) and fitted using a procedure presented by Payne et al.^42^ that allows estimation of the amount of oxygen associated with the organic contaminants as compared to those originating from the SnO_2_ (detailed implementation of this procedure is described in Supporting Information). The amount of organic oxygen estimated to be contributed to the O 1s spectra from that carbon overlayer (“O calc”/Sn of 0.03) is about 3% of the lattice oxygen peak size and does not change significantly during the experiments. Moreover, the O 1s region is well fitted using only the contribution from lattice oxygen, and therefore, this organic oxygen component was not included in the O 1s fit. Finally, the contribution of gas-phase CO to the C 1s region can also be observed during CO exposure (Figure 3f), but no peaks corresponding to CO *adsorbates* were observed to be formed on CO dosing in the C 1s region, possibly due to their exceedingly low density on the surface (if nonzero) and the relatively low signal-to-noise ratio within relevant experimental acquisition times.

#### Reduced Sensor Room Temperature Experiment (R_RT)

Figure 4 (bottom panel) shows the sensor’s resistance as a function of CO pressure at room temperature and the step labels of the experiment “R_RT” (reduced_room temperature). Initially, the sensor’s resistance was relatively low, at approximately 320 Ω, consistent with the reduced character of the surface following the UHV calcination and cooling. Upon the first introduction of CO at 0.1 mbar (step 1), the sensor’s resistance did not change significantly, increasing only up to 330 Ω (response as (resistance in UHV)/(resistance in CO) = 1.03). Increasing the pressure of CO up to 0.5 mbar had little effect on the resistance initially, but halfway through the step, it started decreasing and reached 275 Ω at the end (response 1.16) without stabilizing at a constant value. Further increase in CO pressure up to 1.0 mbar did not result in a visible change in resistance trends, which continued to decrease at a similar rate until CO was evacuated in the step “after”, at which point the resistance stabilized at 185 Ω (response 1.72). Subsequent reintroduction of CO at 0.5 mbar (step “reintro”) had a negligible effect on the sensor’s resistance, which remained at 190 Ω (response 1.67).

The quantification of the corresponding XP spectra is shown in Figure 4 (top panel). The first parameter discussed here is the position of the Sn 3d peak (Sn p.p.), which indicates relative band bending near the surface. Since the binding energy position of peaks in XPS is relative to the Fermi energy in the analyzed volume, a rigid shift in peaks across the spectrum indicates a change in the Fermi energy. As soon as CO was introduced during step 1, the Sn 3d peak shifted to higher binding energy, from 486.85 to 486.95 eV. This downward band bending should correspond to a decrease in resistance; however, the resistance remained relatively unchanged, making the sensor’s Fermi energy shift a less likely explanation. It is also possible that the shift was due to a change in the sample’s work function caused by the introduction of CO; however, no similar effects were observed in the other experiments, so this change could be connected to the environment transition, i.e., cooling in UHV and introduction of CO at room temperature, but would have to result from a unique constellation of (unobservable) adsorbates. Other typical causes of rigid binding energy shifts are far less likely, as charging effects are mitigated by the conductive sample, the temperature is stable, and the instrument has been confirmed to be well calibrated. A comparison of band bending observed in Sn 3d5/2 and O 1s in all steps of all experiments, confirming the shift is rigid, is shown in Figure S9.

Following the increase in CO pressure, there was no significant band bending change, with the peak remaining at 486.95 eV. The same position of the Sn 3d peak was observed during subsequent CO evacuation (step “after”) and reintroduction at 0.5 mbar (step “reintro”). At the same time, the stoichiometry of the surface showed no significant changes during any of the steps. The initial O/Sn ratio of 1.23, lower than the ca. 1.38 we obtained for an oxidized surface (see later) and hence consistent with a more reduced surface, is reproduced consistently in this experiment, with the value varying by only ±0.01 between steps.

Due to the lack of apparent changes in the spectra, no definite determination of the mechanisms of resistance change at play can be made. The only clear indication of changes at the surface is the band bending observed between steps “before” and 1, which could be ascribed to CO adsorbing onto the surface and donating electrons into the conduction band through forming shallow surface donor states, albeit below the detection limit of the XPS measurement (no CO adsorbates observed in the C 1s spectrum). However, that would be expected to result in an immediate change in resistance, which was not observed until the following increase in pressure, and when resistance change finally occurred, no further band bending was observed.^3,5^

#### Reduced Sensor High-Temperature Experiment (R_HT)

**Figure 5:**
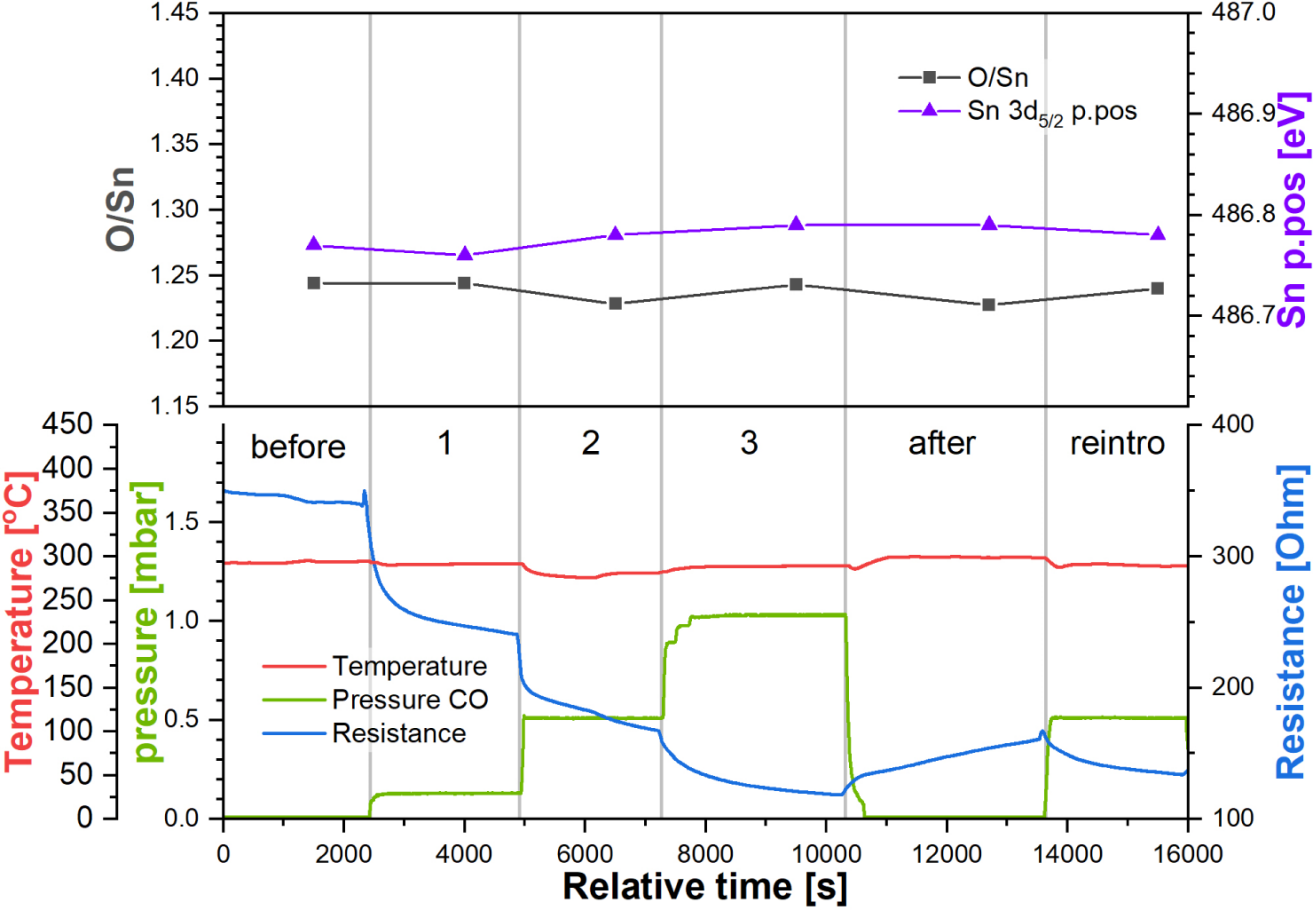
Top panel: quantification of the Sn 3d and O 1s spectra collected during experiment “R_HT”. Bottom panel: temperature and pressure in the NAP cell and sensor’s resistance during experiment “R_HT”. Step labels refer to the CO pressure in the NAP cell (green trace).

Contrary to the previous R-RT experiment, R-HT (reduced_high temperature) was conducted at a typical operating temperature of SnO_2_-based gas sensors of 300 °C. The sensor’s resistance as a function of CO pressure at 300 °C is presented in Figure 5 (bottom panel). The minor temperature fluctuations were caused by the change in CO pressure (and hence the rate of heat dissipation), which required manual adjustments to the heater power output to restabilize the temperature.

The resistance measurements for this reduced sensor started out at 340 Ω during the step “before”. While this value is similar to the initial value (“before”) observed in the R_RT experiment, it is likely coincidental and results from a combination of competing factors, for instance, the treatment the sensor previously received during R_RT (where resistance decreased during the experiment), its subsequent reconstitution in UHV at 400 °C (decreasing number of surface oxygen vacancies as a result of oxygen diffusing from the bulk at elevated temperature, i.e., resistance increase), and the higher sensor operating temperature (increasing number of electronic carriers, i.e., resistance decrease). As soon as the CO was introduced at 0.1 mbar, the resistance began to decrease, eventually reaching 240 Ω (response, *R* = 1.41). Following the increase in the CO pressure in steps 2 (0.5 mbar) and 3 (1.0 mbar), the resistance decreased further to 170 Ω (*R* = 2.00) and 120 Ω (*R* = 2.86), respectively. The removal of CO to UHV increased the resistance only up to 160 Ω (*R* = 2.13), significantly lower than the initial value in UHV. Finally, the reintroduction of CO (0.5 mbar) caused the resistance to decrease again, down to 130 Ω (*R* = 2.63). It is worth noting that the final resistance values of the steps mentioned above are not equilibrium values, as the resistance continued to drift throughout each step, indicating the involvement of a kinetically slow process that does not reach equilibrium within the time frame of a step in this experiment, ca. 2000–3000 s. A similar phenomenon was observed by Kamp et al., who found that oxygen (or vacancy) diffusion in and out of the lattice causes resistance changes over similar time scales.^25^ This phenomenon also accounts for the increase in resistance during the step “after” when CO was removed; without the reducing influence of CO, the vacancy-rich conductive surface is expected to be partially neutralized by the diffusion of oxygen from the bulk at the elevated temperature used. Since the diffusion of oxygen in SnO_2_ is a thermally activated process, the corresponding resistance drift upward is therefore clearly visible here but not in the previously described lower-temperature R_RT experiment.

The quantification parameters based on the corresponding spectra are presented in Figure 5 (top panel). Once again, the decrease in the resistance does not correspond to any significant changes in the XP spectra. The position of the Sn 3d peak remains constant at approximately 486.75–486.80 eV, indicating that no observable band bending occurs near the surface. Similarly, the O/Sn ratio does not vary significantly, remaining between 1.24 and 1.25. Therefore, there are no clear indications of the possible mechanism causing the small resistance change at this temperature.

### Interactions with an O_2_-Oxidized SnO_2_ Surface

#### XP Spectra Fitting

Similar experiments to those described above were conducted on the same sensor, which was regenerated by heating in oxygen and then cooled under the O_2_-atmosphere to the operating temperature (as opposed to cooling under reducing UHV). The sample was brought down to room temperature under 5 mbar O_2_ after 5 h *in situ* calcination at 500 °C at the same pressure of oxygen. These experiments aimed to establish how the CO molecules in the gas phase interact with an oxidized surface of SnO_2_ to produce a sensor response. Once again, the first experiment was conducted at room temperature and the second at 300 °C, following further surface regeneration at 500 °C under 5 mbar O_2_ for 30 min and cooling down to the experimental temperature (300 °C) before O_2_ evacuation.

**Figure 6:**
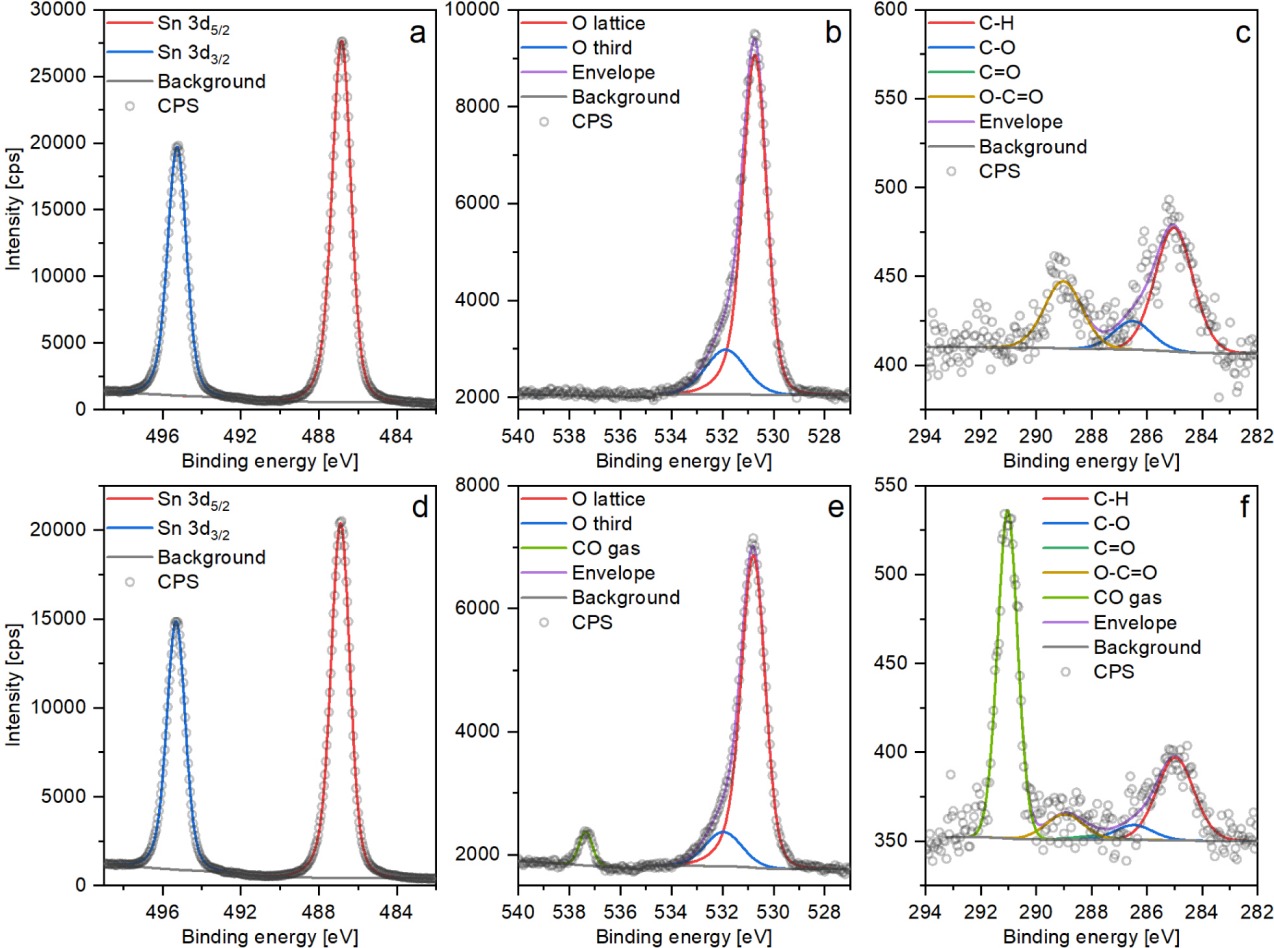
Example XP spectra collected on an O_2_-oxidized SnO_2_ surface during steps “before” (a–c) and “3” (d–f) of the room temperature experiment (O_RT).

Exemplar XP spectra collected on the oxidized surface are presented in Figure 6a–c (after initial preparation—step “before”) and d–f (during 1 mbar CO treatment at room temperature—step 3), while the remaining sets are presented in Supporting Information. Once again, the Sn 3d region only shows contribution from the Sn^4+^ state (Figure 6a,d), and C 1s spectra were fitted using the same model as before to reveal a carbon overlayer with a similar amount of oxygen as before, with an “O calc”/Sn of 0.04. On the other hand, the O 1s region can no longer be modeled using a single peak for the solid phase (Figure 6b). In addition to the major contribution of “O lattice” (which is used to calculate the O/Sn ratio), another smaller peak at ca. 532 eV was included to reproduce the observed photoemission, herein called “O third” for “third-party” oxygen species; its source is discussed below.

#### On the Origin of “O third”

Unlike the “O lattice” peak, the identity of the “O third” cannot be unambiguously determined.^36^ The quantity of organic oxygen estimated to be contributed from carbon species, determined from the C 1s spectrum, could only account for a small portion of the “O third”, and there are no other peaks in the survey spectrum (see Figure S10) that could indicate a counterpart for the excess oxygen to be associated with. Therefore, the oxygen must be bound to itself (molecular O_2_) or to hydrogen, since the latter is undetectable with XPS.

Another possible explanation is that this “O third” signal originates from surface bridging oxygen atoms, i.e., the surface oxygen atoms in SnO_2_ with reduced Sn coordination (from 3 to 2 due to the termination of lattice periodicity). An early in situ XPS study came to this conclusion based on a quantitative spectral analysis and the changes observed upon exposing SnO_2_ to CO.^43^

In the absence of additional information, it is impossible for us to make a positive identification of the origin of this signal, but it should be noted that “O third” signal is not caused by charged chemisorbed oxygen adsorbates as outlined in “ionosorption” models of CGS operation, since the Weisz limit only allows maximum surface densities of 10^–3^–10^–5^ monolayer before adding more adsorbates becomes energetically unfavorable;^26,44^ such low densities are well below the detection limit of XPS, typically estimated at ca. 1% relative atomic abundance.^45^ Considering the available modeling studies,^5,46^ these adsorbates are likely molecular O_2_ bound to the surface oxygen vacancies and therefore, despite having a formal negative charge,^46^ are neutral with respect to the lattice; consequently, these adsorbates do not produce a resistance-increasing double-layer potential near the surface or count toward the Weisz limit.

Even though one would expect a surface treated in O_2_ at a high temperature to be free of vacancies, and hence also free of the molecular O_2_ adsorbates that require a vacancy as an adsorption site, this is not the case for SnO_2_. As explained by Semancik,^47^ when SnO_2_ is cooled down under an O_2_ atmosphere following heat treatment, there exists a temperature range at which spontaneous surface reduction (vacancy formation) is still occurring while the dissociation of O_2_ adsorbates healing the surface slows down, preventing the full healing of the vacancies being created.^47^ Therefore, an appreciable density of vacancies with undissociated O_2_ adsorbates bound to them could remain on the surface after calcination, with the adsorbates contributing to the “O third” component peak. This description is consistent with the work of Semancik and the observations presented in our recent study of oxygen adsorption on SnO_2_.^36^

While surface hydroxyls cannot be excluded entirely as the source of “O third”, we note that any unintentional H_2_O contamination would be at orders of magnitude lower concentration than the intentionally added O_2_ or CO.^8^ However, the effects are typically noticeable on the scale of several percent of relative humidity, i.e., at the hundreds of ppm levels of H_2_O. Considering exposures to up to 1000 ppm of CO and 5000 ppm of O_2_, and the N5.0 purity of the gases, the expected levels of H_2_O are sub-ppm and should not affect the electrical nor spectroscopic measurements. For an additional discussion on the differences between “O third”-type peaks originating from O_2_ and H_2_ adsorption, refer to the Supporting Information of our recent publication.^36^

#### Oxidized Sensor Room-Temperature Experiment (O_RT)

**Figure 7:**
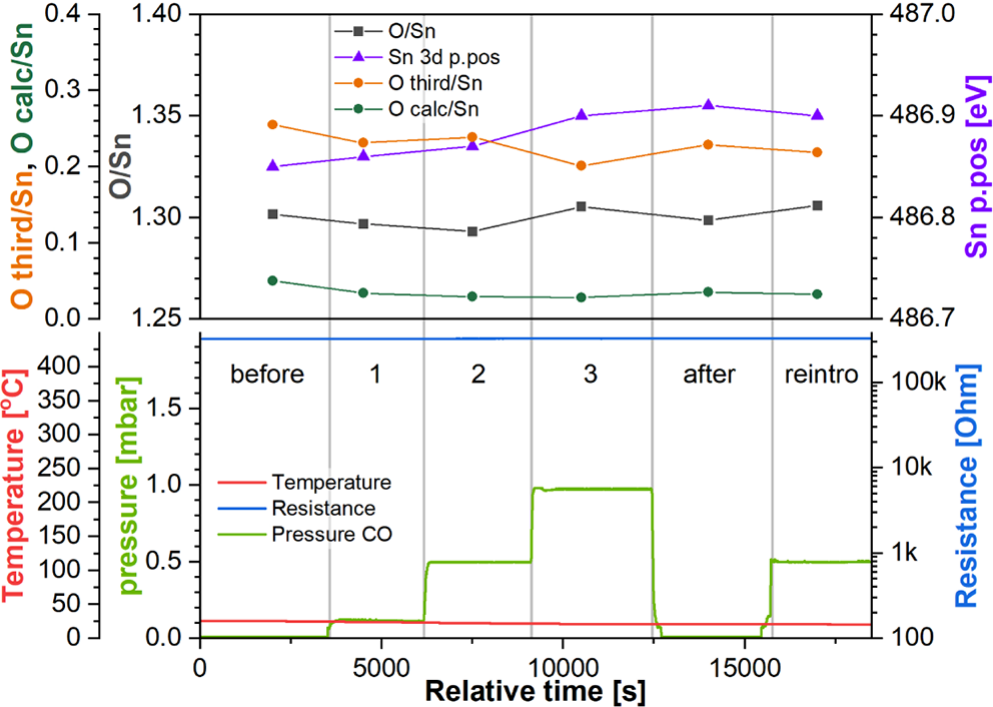
Top panel: sensor’s resistance as a function of CO pressure in the NAP cell at room temperature during experiment “O_RT”. Bottom panel: quantification of the XP spectra collected during experiment “O_RT”. Step labels refer to the CO pressure in the NAP cell (green trace).

The sensor’s resistance as a function of the CO pressure at room temperature is shown in Figure 7 (bottom panel). At this temperature, the oxidized sensor was unresponsive to CO across all pressures, with the resistance measurements remaining constant at 326–328 kΩ (3 orders of magnitude higher than for the reduced sensor). The possible reason for this inertness may be found in the corresponding XP spectra, the quantification of which is presented in Figure 7 (top panel).

The position of the Sn 3d peak did not change significantly during this experiment, remaining between 486.85 and 486.90 eV and indicating no observable band bending. Likewise, the stoichiometry of the surface showed no significant change, with the O/Sn ratio consistently reproduced at between 1.29 and 1.30. We note that this O/Sn ratio, despite being higher than during R_RT and R_HT experiments and indicative of a relatively more oxidized surface, is still below 2, which might be expected for stoichiometric fully oxidized SnO_2_. The variance we see from this value is attributed to our use of standard relative sensitivity factors for quantification rather than experimentally derived values from an SnO_2_ standard, as opposed to an indication of the absolute degree of reduction. Given that SnO_2_ starts to spontaneously evolve O_2_ at temperatures above 130 °C, and vacancy healing by ambient O_2_ dissociation and incorporation into the SnO_2_ lattice is a thermally activated process,^47^ the relatively low pO_2_ during surface oxidation (5 mbar) may also be insufficient to fully oxidize the surface. Hence, the surface is unlikely to be fully stoichiometric, although we assume that our highest observed O/Sn ratio of ca. 1.38 (see below) should be considered close to stoichiometric.

Finally, the “O third” component also remains relatively constant, although it shows more variation than the other parameters. An estimate of organic oxygen based on the C 1s spectra (“O calc”) was included in the plot to show that the “O third” component, whose area was much larger than “O calc” during every step, cannot be explained by the adventitious carbon contamination. Initially at 0.26, “O third” gradually decreases down to 0.22 over the course of this experiment, indicating that some of the oxygen adsorbates may have been removed from the surface, possibly by reacting with the CO gas molecules. However, the limited change in “O third”, together with the lack of change in stoichiometry and band bending, indicates that any reaction of CO with oxygen surface atoms and preadsorbed O_2_ must be exceedingly slow at this temperature.

#### Oxidized Sensor High-Temperature Experiment (O_HT)

**Figure 8:**
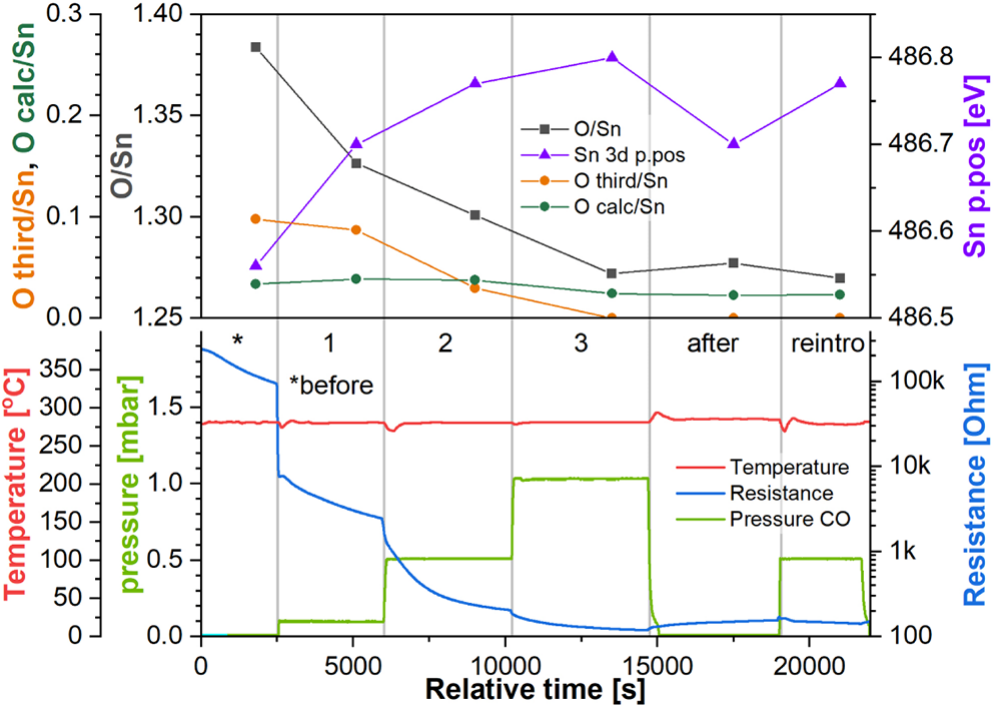
Top panel: quantification of the XP spectra collected during experiment “O_HT”. Bottom panel: Ssensor’s resistance as a function of CO pressure at 300 °C during experiment “O_HT”. Step labels refer to the CO pressure in the NAP cell (green trace).

The second part of investigating CO adsorption onto an oxidized SnO_2_ surface was performed at 300 °C after reconstituting the surface in 1 mbar O_2_ at 300 °C for 15 min and evacuating to UHV prior to the experiment (“before”). Subsequently, the CO was introduced into the NAP cell, resulting in significant resistance changes, as shown in Figure 8 (bottom panel).

In this experiment, the initial resistance (at the end of step “before”) had decreased to 90 kΩ. As soon as the CO was introduced, the sensor’s resistance dropped rapidly to 7.6 kΩ (vs 90 kΩ, the final value from step “before”, response 11.9) and then gradually down to 2.5 kΩ (*R* = 35.7) by the end of step 1. Following the increase in the CO pressure in the next two steps to 0.5 and then 1.0 mbar, the resistance decreased to 205 Ω (*R* = 435) and 120 Ω (*R* = 770) by the end of steps 2 and 3, respectively. The evacuation of CO to UHV during step “after” led to a resistance increase only up to 155 Ω (*R* = 588), a value lower by a factor of 580 than that from the end of step “before” (90 kΩ), indicating that the sensor’s resistance decreased permanently due to its interaction with CO in the absence of oxygen in the ambient. The final introduction of CO during step “reintro” resulted in the resistance decreasing again, eventually down to 140 Ω (*R* = 643). Consequently, once the surface has been reduced, CO has a limited effect on the resistance, confirming the relatively invariant resistance measured during the R_RT and R_HT experiments.

The quantification of the corresponding XP spectra presented in Figure 8 (top panel) shows significant changes in band bending as soon as the CO was introduced into the NAP cell. The initial position of Sn 3d at 486.55 eV is relatively low and consistent with a highly oxidized surface. Subsequent CO exposure during steps 1, 2, and 3 resulted in the Sn 3d peak moving to 486.70 (+0.15 eV), 486.75 (+0.20 eV), and 486.80 eV (+0.25 eV), respectively, indicating downward band bending; both this and the concurrently decreasing resistance of the sensor are consistent with surface reduction. When CO was evacuated during step “after”, the position of the peak shifted to 486.70, indicating upward band bending (i.e., unbending of the bands) of 0.10 eV relative to the previous step (step 3). This change, consistent with the slight increase in resistance, could be explained either by the desorption of molecular CO, which would remove surface electron donor states, or surface oxygen vacancy diffusion into the bulk beyond the near-surface region (lattice oxygen diffusion to the surface) where the oxygen vacancies can act as electron donors (effectively healing of the surface by diffusion of bulk oxygen ions). Finally, the reintroduction of CO caused the bands to bend downward again, with the Sn 3d peak shifting to 486.75 eV, again consistent with the lower resistance observed during that step.

In addition to the evident band bending, the O/Sn ratio also decreased significantly, indicating that the surface stoichiometry changes were substantial enough to be detected using this instrument. The initial O/Sn ratio, at 1.38, is larger than the O/Sn ratio during the previous experiment (O_RT), attributable to the surface being oxidized immediately prior to the experiment rather than being allowed to reduce slightly during the lengthy cooling down to room temperature (as was the case for O_RT) according to the mechanism proposed by Semancik et al.^47^ Shortly after CO introduction, the surface started reducing, with the O/Sn ratio decreasing to 1.33, 1.30, and 1.27 during steps 1, 2, and 3, respectively. To re-emphasize, the O/Sn ratio compares the relative abundance of only lattice oxygen to tin ions and does not include any other oxygen species like adsorbates or organic surface contaminants, and therefore is a measure of the lattice stoichiometry rather than total oxygen content in the analyzed volume. This change in the O/Sn ratio is also reflected in the resistance measurements, which show a marked decrease with each step of progressing surface reduction. After CO evacuation, the O/Sn ratio remained largely unchanged, consistent with the small change in resistance accompanying it and with the surface being already reduced at the start of this step, which was shown to limit further reaction of surface oxygen atoms with CO molecules during “R_HT”, where the O/Sn ratio changed by a similar amount during the experiment.

In addition to the changes observed in the O/Sn ratio, the “O third” peak also decreased in intensity. It is unclear whether the peak disappears due to the high temperature and the absence of ambient O_2_, or if the CO reacts with these oxygen adsorbate species following the Eley–Rideal mechanism. The reaction of CO with preadsorbed oxygen has been shown to be possible in computational studies.^2−5^ Therefore, both the spontaneous desorption of preadsorbed O_2_ and Eley–Rideal oxidation of CO likely play a role in removing the “O third” signal. On the other hand, the peak cannot be ascribed to adventitious carbon contamination due to the mismatch in trends observed in “O third” and “O calc”. The latter remains constant, and similar to the reduced surface experiments at “O calc”/Sn of 0.03, the former increases from 0 to 8 times more than “O calc” and back to 0, far beyond the apparent precision of this method.

The changes observed in the O/Sn and “O third”/Sn ratios were significantly larger than during any of the previous experiments, and so were the changes in resistance; i.e., consumption of lattice oxygen by reaction with CO and/or desorption of oxygen adsorbates could be responsible for the resistance change. However, it is worth re-emphasizing here that “O third” cannot be the charged oxygen adsorbates invoked in ionosorption (*q.v.* our previous discussion regarding the Weisz limit), which corresponds with observations we made previously for the interaction of oxygen with the surface of SnO_2_.^36^ Similarly to there, we note that any truly “ionosorbed” oxygen species would likely be below the detection limit of our experiment, and hence we cannot comment on their presence or any changes in their concentration. But as we previously concluded, we see no need to invoke these additional species when the behavior we observe can be directly explained by the changes in oxygen vacancy concentration we have measured. Consequently, we conclude the formation of surface oxygen vacancies in SnO_2_ via the removal of surface lattice oxygen atoms during CO oxidation plays a vital role in producing sensor response toward this target gas.

## Conclusions

This article reports the results of a joint macro- and spectroscopic analysis of the interactions between a model SnO_2_-based sensor and CO by using NAP XPS with simultaneous resistance measurements. The experiments performed on a reduced and oxidized surface at room temperature and 300 °C reveal that the magnitude of sensor response depends not only on the sensor’s temperature but predominantly on the degree of initial surface oxidation.^36^ While the response to equivalent pressures of CO was enhanced by the increase in temperature on both the reduced and oxidized surfaces, the resistance change at high temperature was over 2 orders of magnitude larger on the oxidized surface compared to the reduced, decreasing from 90,000 Ω to only 120 Ω (response, *R* = 750). This difference in responsivity was also visible in the accompanying XP spectra, which show a substantial decrease in the near-surface O/Sn stoichiometry in contrast to the other experiments, which showed little to no change in both O/Sn stoichiometry and resistance. When the starting point of detection is an oxidized surface, one that has a higher availability of surface lattice oxygen atoms that can react with CO and large initial resistance, the exposure to CO can lead to a larger change in both quantities before the surface oxygen atoms are depleted. On a reduced surface, however, the change can only be as large as permitted by the diffusion of bulk lattice oxygen atoms to the surface where they can react with CO. Consequently, these results constitute clear evidence of the involvement of near-surface vacancies in the CO detection mechanism of SnO_2_-based sensors.

The origin of “O third” in this experiment is speculative and requires further investigation. Experiments with O_2_/CO/H_2_O exchange and co-dosing could reveal subtle differences between the shape of the O 1s asymmetry, allowing more definite determination or even resolution of the species’ contributions to the “O third” peak intensity. This is of particular interest to the community, considering that humidity is ubiquitous in almost any sensor environment and has complicated interactions with the surface of SnO_2_; it forms rooted and terminal hydroxyls^48^ through reactions with both lattice oxygen and oxygen vacancies that have opposing effects on the surface’s conductivity and healing vacancy sites, altering the surface’s adsorption sites and the free electron gas.

While the direct reduction of the surface of SnO_2_ by CO is clearly the major contributor to resistance change, the other proposed mechanisms, such as the donation of electrons by CO upon adsorption, cannot be disproved based on the results presented above. Moreover, some of the data shown in this study, like the change in resistance during experiment R_RT and the complete lack of response in O_RT, seem better explained by such electron-donating interaction. In the former case, the temperature should be too low to enable surface reduction by CO and repopulation of surface oxygen lattice sites to enable the reduction, yet some change in resistance was registered. In the latter case, the lack of change could be explained by preadsorbed O_2_ occupying all of the available adsorption sites, preventing CO from interacting with the surface. Taken together with our previous work on the correlation of resistance change in SnO_2_ with surface oxygen vacancy concentration and consequent band bending,^36^ this article then provides a complete picture of how gas sensitivity in SnO_2_-based sensors toward both oxidizing and reducing gases can be described based only on the variation of surface oxygen vacancy concentration.

## Supporting Information Available

Additional experimental details; XP spectra processing; complete set of XP spectra; and XPS survey scan of the oxidized surface (PDF)

## Abbreviations

CGS: conductometric gas sensor
NAP: near-ambient pressure
XPS: X-ray photoelectron spectroscopy
