# Supplementary material for: Interplay between CO and Surface Lattice Oxygen Ions in the Vacancy-Mediated Response Mechanism of SnO2-Based Gas Sensors
Source: ACS Sens. 2025 Mar 1;10(3):1898–908. doi: 10.1021/acssensors.4c03047 (PMC11959589; doi:10.1021/acssensors.4c03047)
Supplement: Supplementary file 1 — se4c03047_si_001.pdf [file se4c03047_si_001.pdf]

Electronic Supporting Information

Interplay Between CO and Surface Lattice Oxygen Ions in Vacancy-Mediated Response Mechanism of SnO_2-based Gas Sensors

Stefan Kucharski, Michael Vorochta, Lesia Piliai, Andrew M. Beale and *Christopher Blackman

Contents

Additional Experimental Details S1
XP spectra processing S1
Reduced surface XP spectra S4
Experiment R_RT S4
Experiment R_HT S6
Oxidised surface XP spectra S8
Experiment O_RT S8
Experiment O_HT S10
Rigid shift in peak positions – the relationship between Sn 3d and O 1s peaks S12
Oxidised surface experiments – origin of 'O third' S13
References S13

Additional Experimental Details

The SPECS NAP XPS system used in this study comprises a monochromated Al Kα X-ray source (1486.7 eV) with a spot size of between 400 and 500 µm; the X-ray beam is oriented at a magic angle to the analyser axis, thus eliminating the angle-dependence of photoemission intensity.

In these experiments, the XP spectra were collected using the following parameters: survey scans – 60 eV pass energy, 100 ms dwell time, 0.5 eV step; high-resolution Sn 3d, O 1s and C 1s scans – 20 eV pass energy, 400 ms dwell time, 0.05 eV step. The number of scans per region was incremented with increasing analysis pressure to counteract the intensity loss due to photoelectrons scattering off the gas molecules.

XP spectra processing

The XP spectra presented in this work were quantified using CasaXPS.[1] The binding energy scale of the spectra was not adjusted due to a lack of a reliable internal reference, with the Sn and O peaks expected to shift in response to atmosphere changes and C 1s not being a reliable reference.[2] However, the instrument's calibration was confirmed prior to the

experiments using the Au 4f peak, whose position was observed at 83.98 eV, very close to the literature value of 84.0 eV for bulk Au atoms.[3]

The Sn 3d spectra were assigned a Tougaard background, which reproduced well the shape of the photoemission outside of the peaks and provided a sound basis for a reproducible quantification, as opposed to the Shirley method, which overestimated the background intensity in some instances, introducing a random error into the quantification. The spectra were fitted with two components based on an asymmetric pseudo-Voigt (convoluted Gaussian-Lorentzian) lineshape, whose parameters were adjusted to faithfully reproduce the first Sn 3d spectrum in each experiment and subsequently were kept unchanged to make any peak shape change more evident. The position, FWHM and area of the two components were unconstrained and fitted to the recorded spectra using the Marquardt algorithm.

The O 1s spectra were processed in a similar fashion, except, in this instance, the Shirley algorithm, a default choice for such spectra, proved to yield consistent results and therefore was applied. In the case of UHV-reduced samples, this single component was sufficient to reproduce faithfully the recorded photoemission. An analogous asymmetric pseudo-Voigt lineshape moulded to the first O 1s spectrum in an experiment was used to reproduce the photoemission attributable to the lattice oxygen atoms of SnO_2. However, fitting of the spectra recorded on the O_2-oxidised surface required an additional component. Since this component, named ‘O third’ for ‘third-party species’, cannot be unambiguously identified and likely corresponds to a mixture of various species, including oxygen adsorbates, organic contaminants and surface hydroxyls, it was assigned a generic, symmetric Voigt lineshape. Due to the presence of ‘O third’, the ‘O lattice’ lineshape could not be moulded to the first recorded O 1s photoemission and instead spectrum collected during step ‘after’ of experiment O_HT was used since it was also collected in UHV and could be fitted with a single asymmetric peak. As in the case of Sn 3d fitting, the binding energy position, FWHM and area of the peaks remained unconstrained to maximise the information extractable from the spectra, and the fitting to the recorded data was performed using the Marquardt algorithm.

Finally, the C 1s spectra were also assigned a Shirley background and were fitted with four peaks. The main peak located at about 285 eV corresponds to C-H and C-C carbon. According to the procedure published by McIntyre et al.,[4] the subsequent three peaks, corresponding to C-O, C=O and O-C=O carbon were constrained to binding energy position of +1.5 eV, +3.0 eV and +4.0 eV, respectively. Such processing allows a rough estimation of the amount of oxygen associated with the organic contamination on the surface and therefore helps validate the O 1s spectra peak fitting.

Further processing of the spectra involved calculating the relative atomic abundances of the three elements. The areas of the peaks were obtained from the models described above and normalised with respect to their respective relative sensitivity factors (RSF) based on the

Scoffield cross-sections[5]; the values of 25.05, 2.93 and 1 were used for Sn 3d (the sum of the 5/2 and 3/2 peaks), O 1s and C 1s, respectively.

Subsequently, the RSF-normalised peak areas were normalised again, this time with respect to the energy-dependent attenuation of photoelectrons by the gas in the NAP cell of the spectrometer. The full description of this normalisation procedure, including its derivation, is presented elsewhere[6]; however, it can be summarised as follows: the attenuation normalisation method employs empirically-derived relationships between the intensity of Au 4f peaks and the kinetic energy of photoelectrons (modulated by changing the incident X-ray energy) at a series of pressure levels (UHV, 0.1 mbar, 0.5 bar, 1 mbar and 2 mbar) to establish coefficients that normalise the XPS intensity to what it would be if collected in UHV. This method allows more accurate determination of the O/Sn and C/Sn ratio, which would otherwise be underestimated due to the O 1s and C 1s electrons (which have a lower kinetic energy than Sn 3d at the same excitation energy) being attenuated more strongly than Sn 3d in non-UHV conditions, leading to lower peak intensity. Such normalisation procedure was applied to both the 'O lattice' and 'O third' peaks in determining the O/Sn and 'O third'/Sn ratio, as well as for calculating the 'O calc' from the C 1s spectra.

'O calc' estimation is based on the fitting of the C 1s spectrum proposed by McIntyre et al. and outlined above, where the C 1s spectrum is fitted with four components, C-H, C-O-C, C=O and O-C=O, corresponding to increasingly more oxidised carbon atoms. The area of each component, following applicable normalisations, was multiplied by the stoichiometric ratio of oxygen associated with it (0, 0.75, 1 and 1.5, respectively, see the original paper for justification) and added together to obtain a rough estimate of the amount of oxygen associated with the adventitious carbon overlayer.

Following the two normalisation procedures, the areas of 'O lattice', O third' and 'O calc' were divided by the area of the Sn 3d peaks to obtain O/Sn, 'O third'/Sn and 'O calc'/Sn, which are the values presented in the manuscript.

Reduced surface XP spectra

Experiment R_RT

Below are Figures S1 and S2, presenting all the spectra collected in experiment R_RT. High-resolution scans of Sn 3d, O 1s and C 1s were collected at every step. The names of the steps above each set of spectra correspond to those presented in the manuscript; the details regarding the temperature and pressure during spectra acquisition are outlined therein.

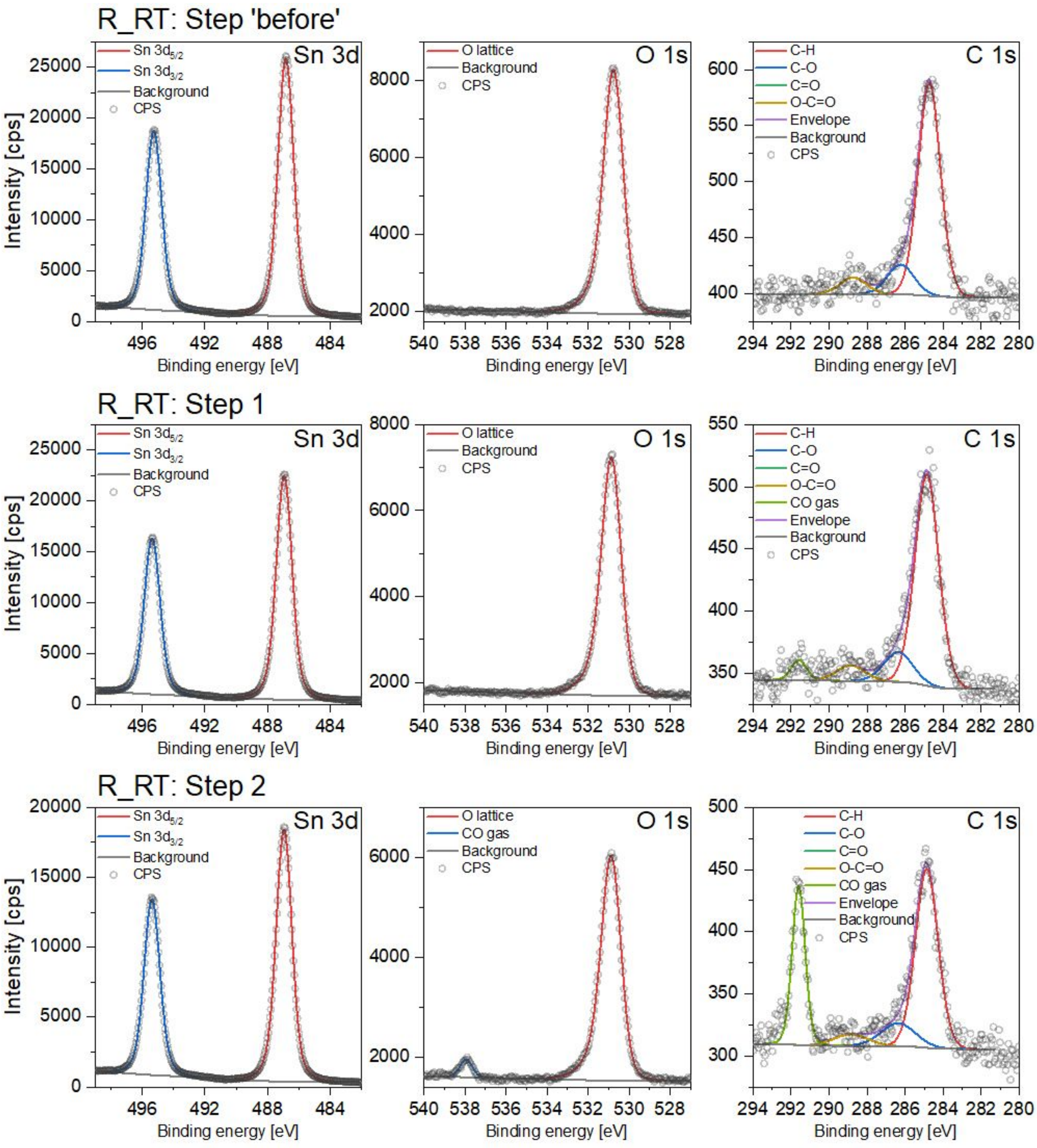

Figure S1: XP spectra collected during experiment R_RT: (top-to-bottom) Steps 'before', 1 and 2. (left-to-right) Sn 3d, O 1s and C 1s.

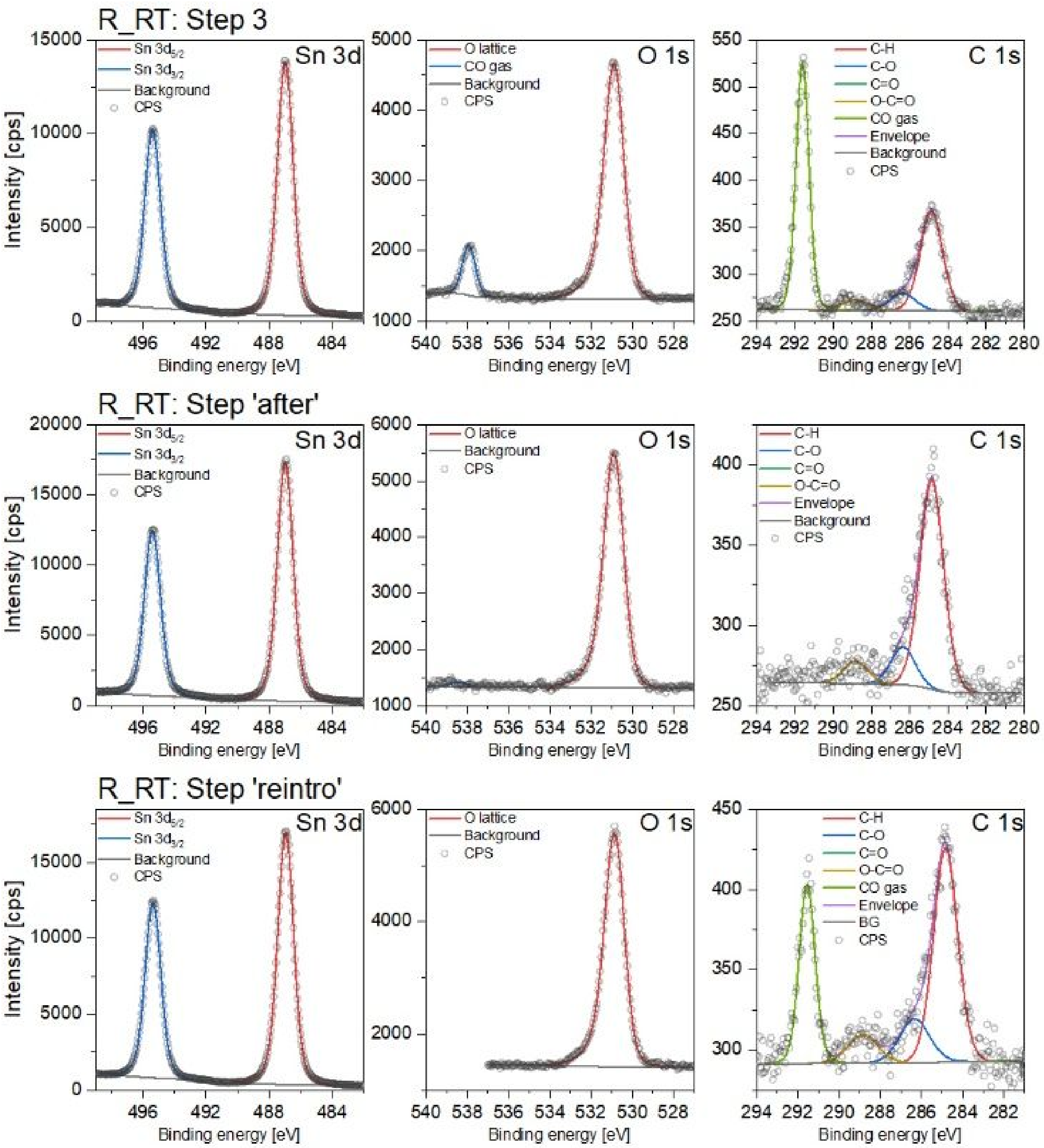

Figure S2: XP spectra collected during experiment R_RT: (top-to-bottom) Steps 3, 'after' and 'reintro'. (left-to-right) Sn 3d, O 1s and C 1s.

Experiment R_HT

Below are Figures S3 and S4, presenting all the spectra collected in experiment R_HT. High-resolution scans of Sn 3d, O 1s and C 1s were collected at every step. The names of the steps above each set of spectra correspond to those presented in the manuscript; the details regarding the temperature and pressure during spectra acquisition are outlined therein.

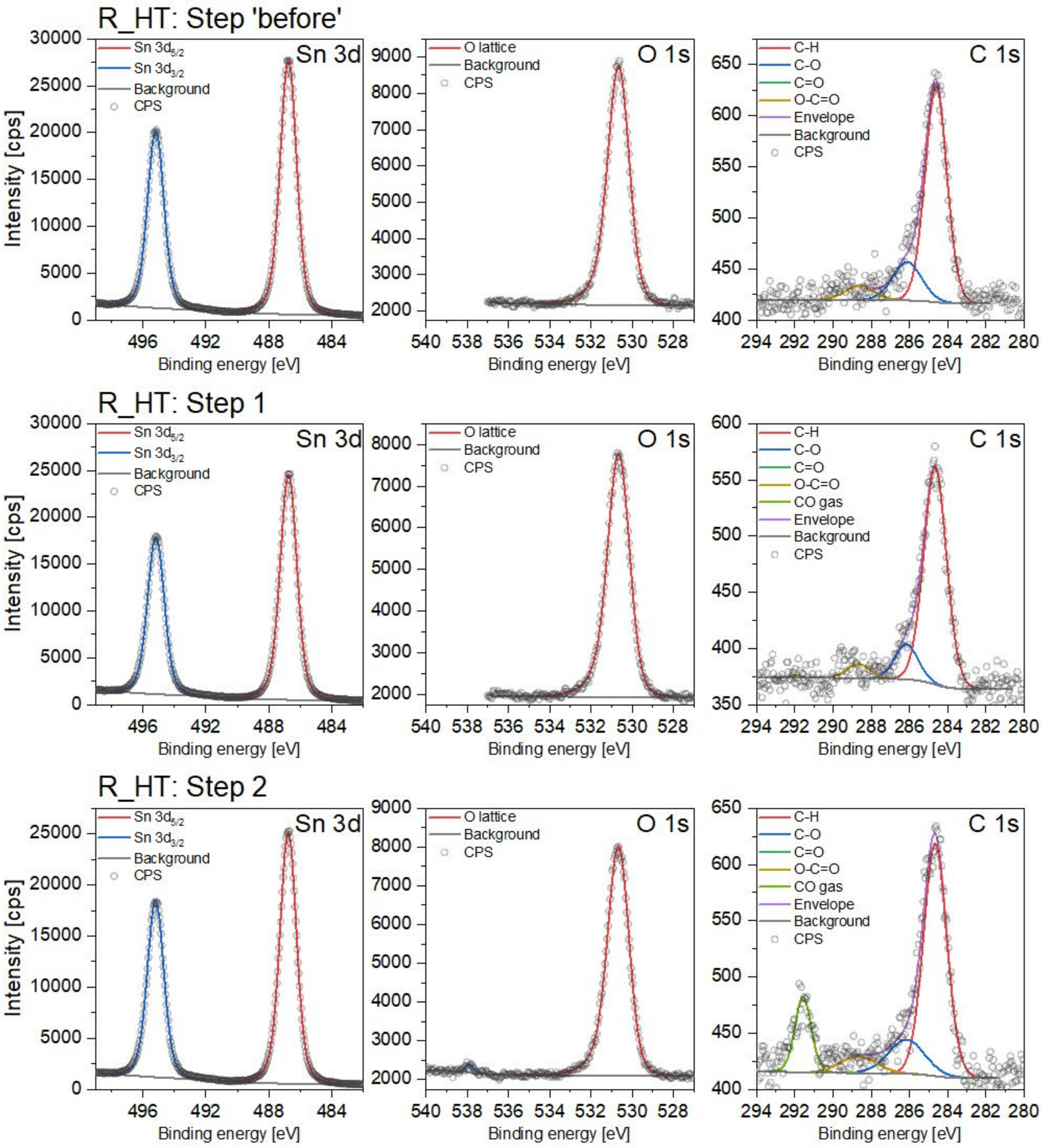

Figure S3: XP spectra collected during experiment R_HT: (top-to-bottom) Steps 'before', 1 and 2. (left-to-right) Sn 3d, O 1s and C 1s.

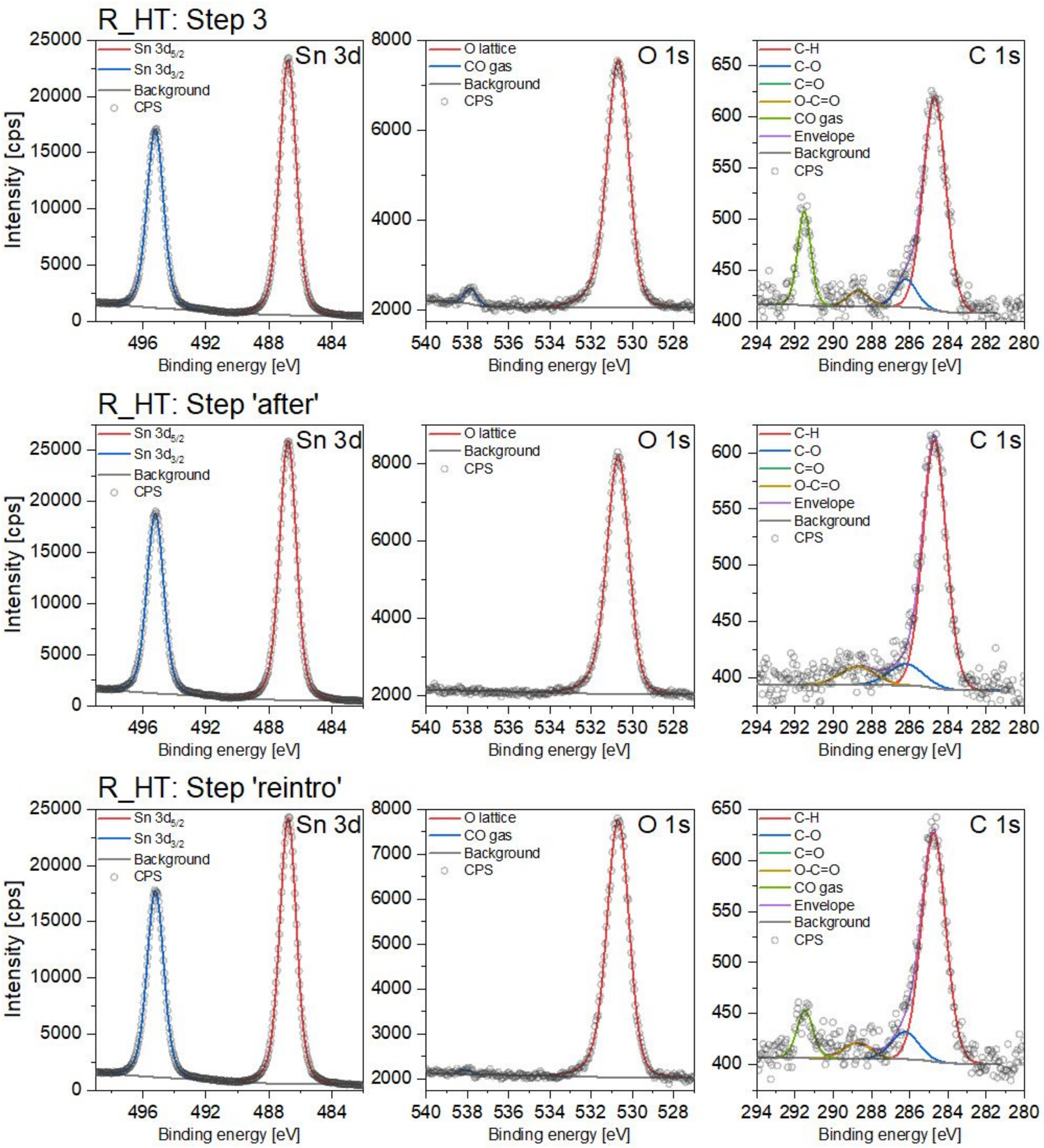

Figure S4: XP spectra collected during experiment R_HT: (top-to-bottom) Steps 3, 'after' and 'reintro'. (left-to-right) Sn 3d, O 1s and C 1s.

Oxidised surface XP spectra

Experiment O_RT

Below are Figures S5 and S6, presenting all the spectra collected in experiment O_RT. High-resolution scans of Sn 3d, O 1s and C 1s were collected at every step. The names of the steps above each set of spectra correspond to those presented in the manuscript; the details regarding the temperature and pressure during spectra acquisition are outlined therein.

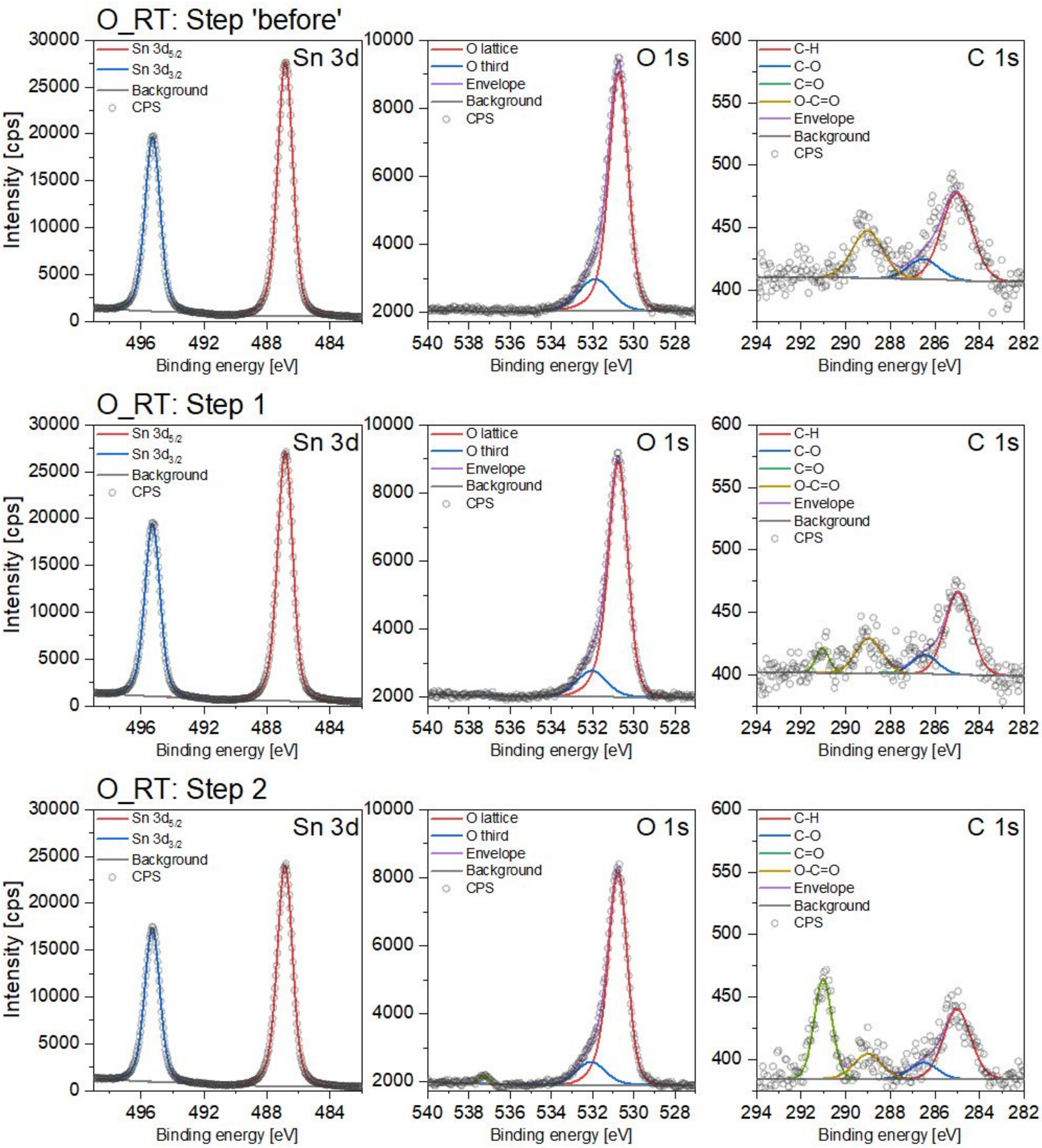

Figure S5: XP spectra collected during experiment O_RT: (top-to-bottom) Steps 'before', 1 and 2. (left-to-right) Sn 3d, O 1s and C 1s.

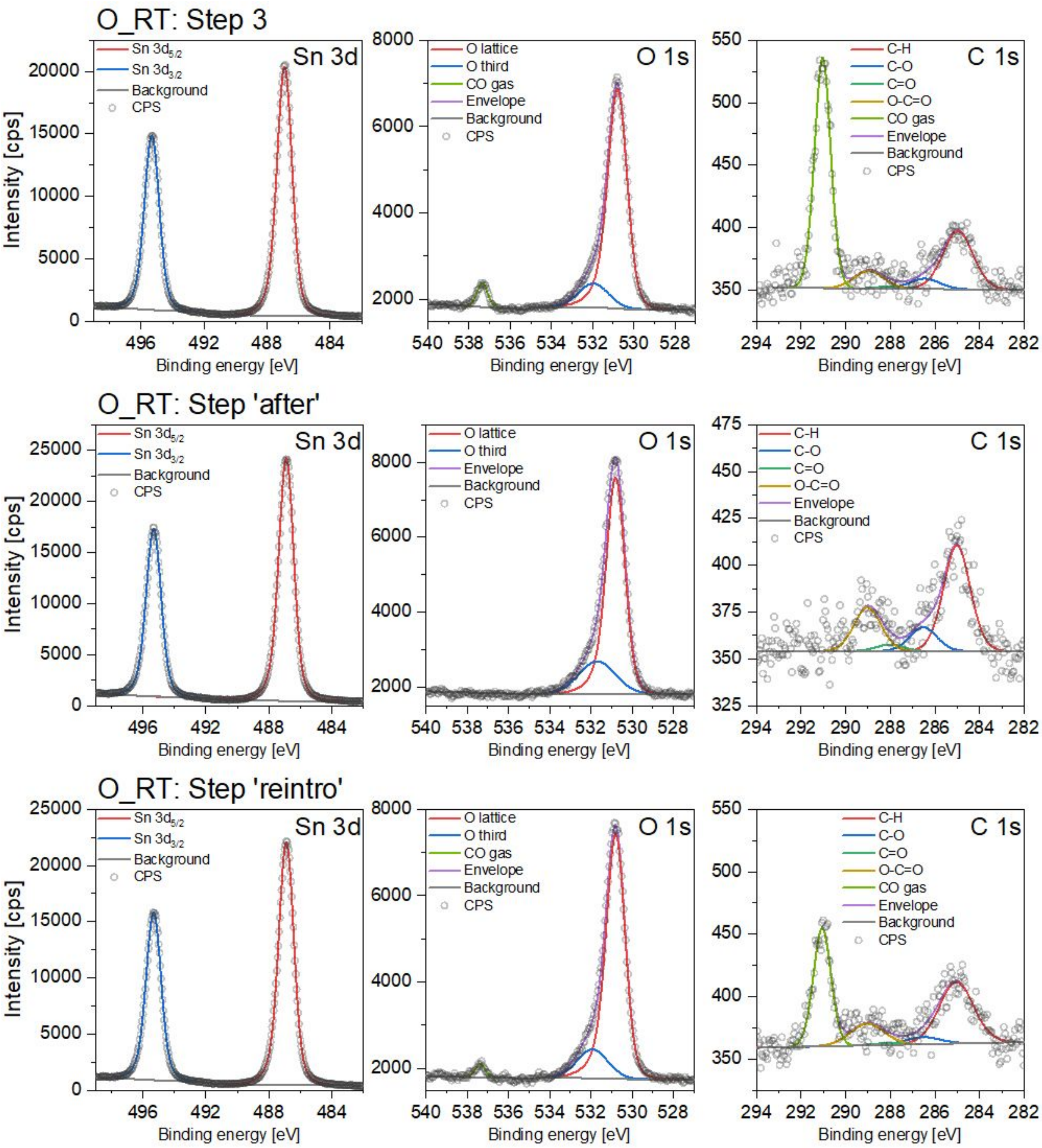

Figure S6: XP spectra collected during experiment O_RT: (top-to-bottom) Steps 3, 'after' and 'reintro'. (left-to-right) Sn 3d, O 1s and C 1s.

Experiment O_HT

Below are Figures S7 and S8, presenting all the spectra collected in experiment O_HT. High-resolution scans of Sn 3d, O 1s and C 1s were collected at every step. The names of the steps above each set of spectra correspond to those presented in the manuscript; the details regarding the temperature and pressure during spectra acquisition are outlined therein.

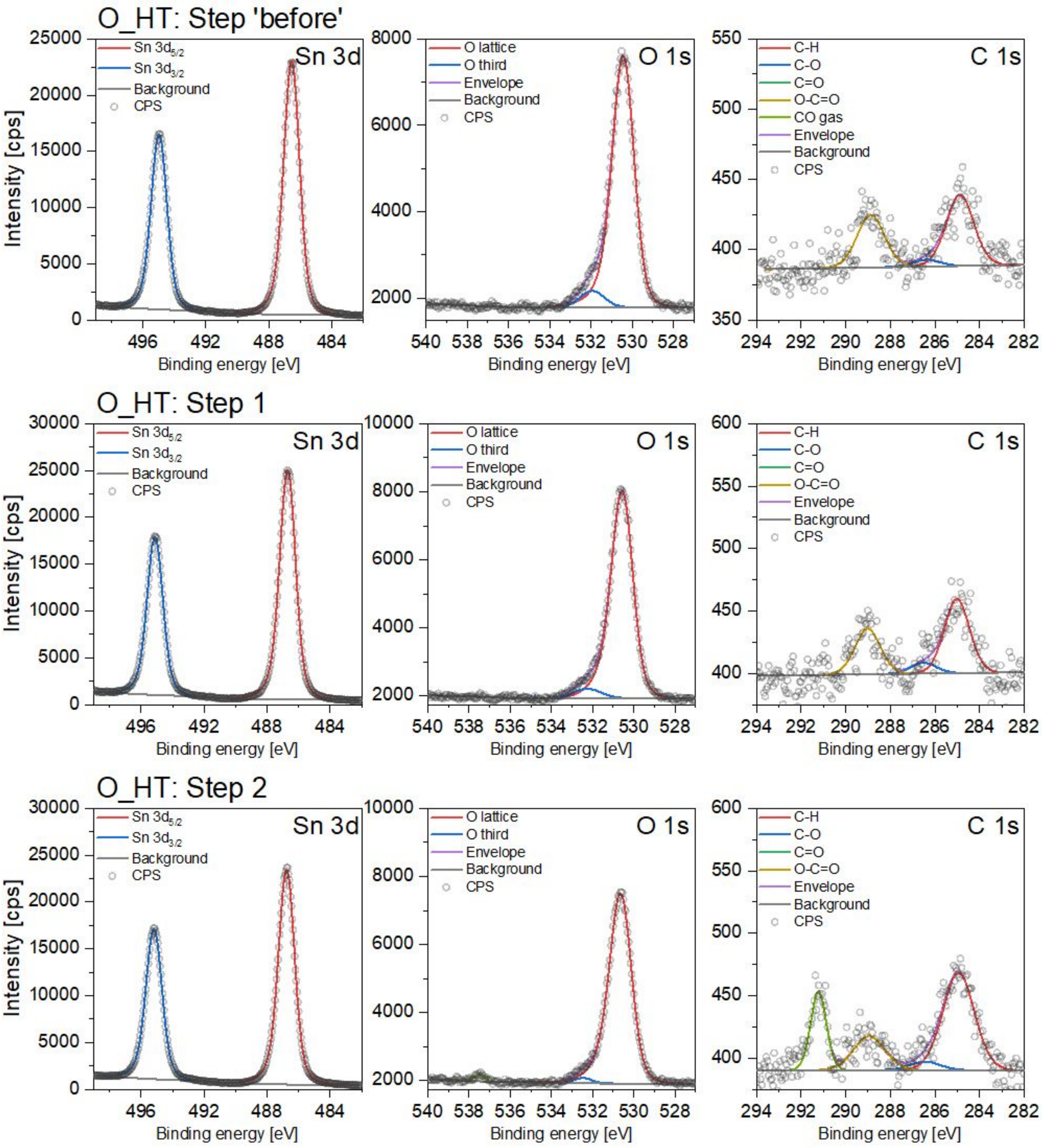

Figure S7: XP spectra collected during experiment O_HT: (top-to-bottom) Steps 'before', 1 and 2. (left-to-right) Sn 3d, O 1s and C 1s.

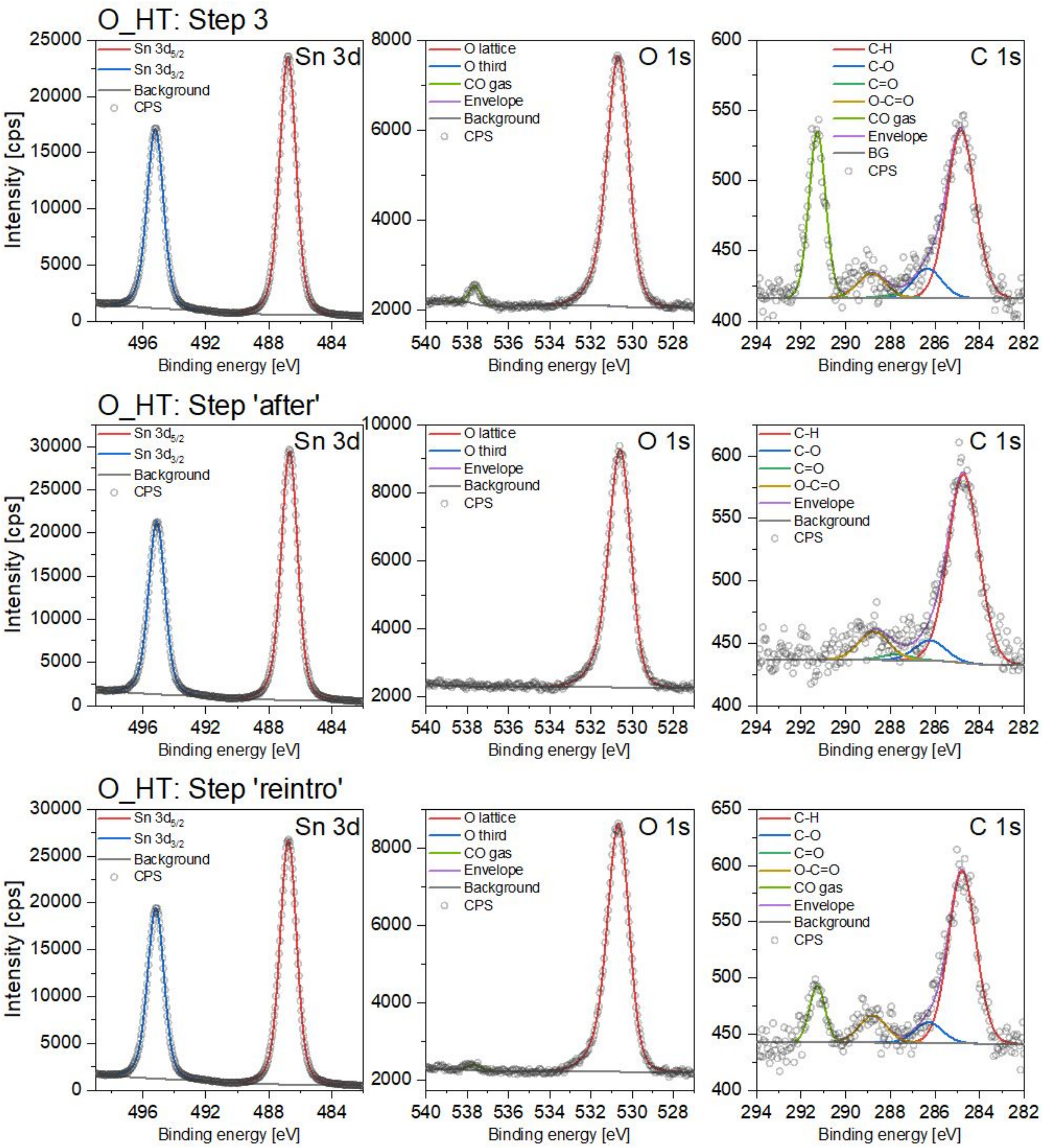

Figure S8: XP spectra collected during experiment O_HT: (top-to-bottom) Steps 3, 'after' and 'reintro'. (left-to-right) Sn 3d, O 1s and C 1s.

Rigid shift in peak positions – the relationship between Sn 3d and O 1s peaks

Figure S9 shows the relationship between the O 1s and Sn 3d peak positions in order to confirm whether the peak shift observed in Sn 3d is due to a rigid shift across all spectrum, which would indicate a change in the Fermi level of the sample, or a change in oxidation state, which would not affect the peak position of the O 1s. The fact that all the points align on the diagonal, the shift in the spectrum is rigid and the observed changes are due to a change in the Fermi level, not the oxidation state of Sn.

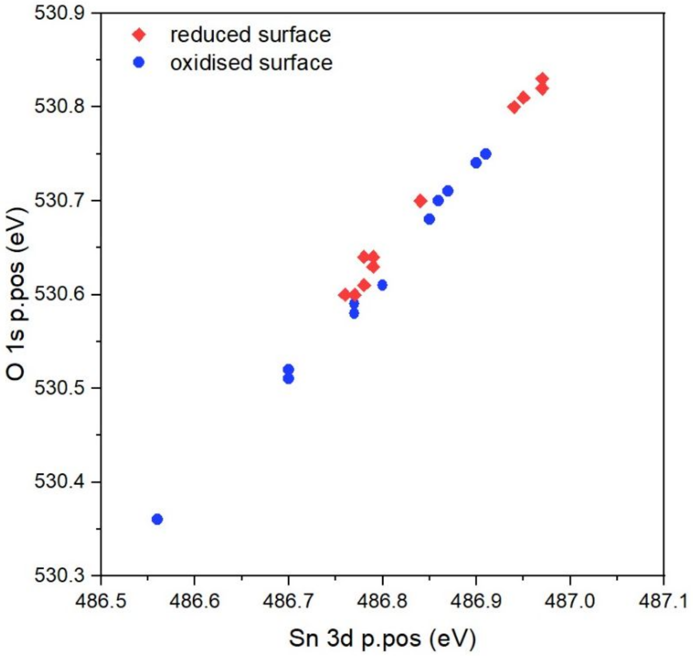

Figure S9: A scatter plot showing the position of the O 1s peak versus the Sn 3d 5/2 peak for pairs from spectra taken in all reduced (red diamonds) and oxidised (blue circles) experiment steps.

Oxidised surface experiments – origin of ‘O third’

Figure S10 presents one of the survey spectra collected during experiments on the O_2-oxidised surface of the sample sensor. All the peaks visible in the spectrum can be assigned to Sn, O and C indicating that other than the typical small carbon overlayer there are no contaminants present in the investigated volume. Consequently, there are no species with which the ‘O third’ signal could be associated, indicating again that it most likely source is elemental oxygen – O_2.

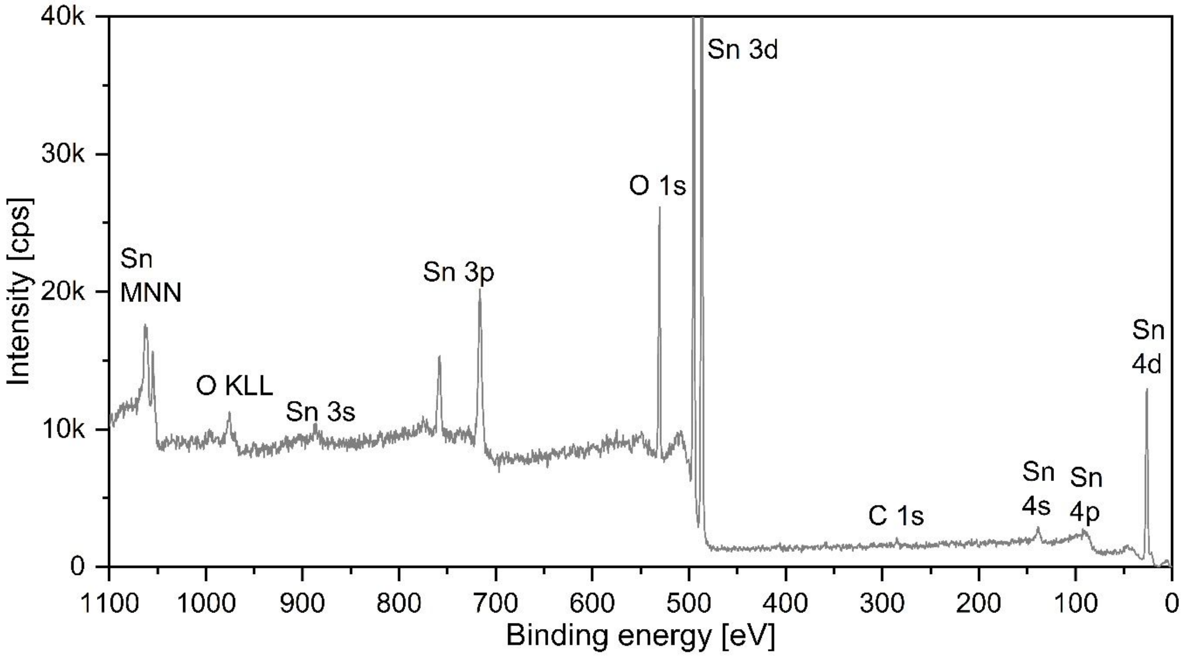

Figure S10: Example survey spectrum collected during experiment O_HT, step ‘before’. The spectrum shows that only O and Sn are present in appreciable quantities along with a minute amount of carbon.

References

(1) Fairley, N.; Fernandez, V.; Richard-Plouet, M.; Guillot-Deudon, C.; Walton, J.; Smith, E.; Flahaut, D.; Greiner, M.; Biesinger, M.; Tougaard, S.; Morgan, D.; Baltrusaitis, J. Systematic and Collaborative Approach to Problem Solving Using X-Ray Photoelectron Spectroscopy. *Applied Surface Science Advances* **2021**, *5*, 100112. https://doi.org/10.1016/j.apsadv.2021.100112.

(2) Greczynski, G.; Hultman, L. Compromising Science by Ignorant Instrument Calibration—Need to Revisit Half a Century of Published XPS Data. *Angewandte Chemie* **2020**, *132* (13), 5034–5038. https://doi.org/10.1002/ange.201916000.

(3) Citrin, P. H.; Wertheim, G. K.; Baer, Y. Core-Level Binding Energy and Density of States from the Surface Atoms of Gold. *Phys Rev Lett* **1978**, *41* (20), 1425–1428. https://doi.org/10.1103/PhysRevLett.41.1425.

(4) Payne, B. P.; Biesinger, M. C.; McIntyre, N. S. X-Ray Photoelectron Spectroscopy Studies of Reactions on Chromium Metal and Chromium Oxide Surfaces. *J Electron Spectros Relat Phenomena* **2011**, *184* (1–2), 29–37. https://doi.org/10.1016/j.elspec.2010.12.001.

(5) Scofield, J. H. *Theoretical Photoionization Cross Sections from 1 to 1500 KeV*; 1973. https://doi.org/https://doi.org/10.2172/4545040.

(6) Kucharski, S.; Ferrer, P.; Venturini, F.; Held, G.; Walton, A. S.; Byrne, C.; Covington, J. A.; Ayyala, S. K.; Beale, A. M.; Blackman, C. Direct in Situ Spectroscopic Evidence of the Crucial Role Played by Surface Oxygen Vacancies in the O_2-Sensing Mechanism of SnO_2. *Chem Sci* **2022**, *13* (20), 6089–6097. https://doi.org/10.1039/D2SC01738E.
